# In silico prediction and in vitro validation of the effect of pH on adhesive behaviour of the fused CsgA-MFP3 protein

**DOI:** 10.1186/s13568-022-01435-5

**Published:** 2022-07-15

**Authors:** Keyvan Shahryarimorad, Atefeh Alipour, Yousof Saeedi Honar, Behrouz Abtahi, Mohammad Ali Shokrgozar, Hosein Shahsavarani

**Affiliations:** 1 Laboratory of Regenerative Medicine and Biomedical Innovations, Pasteur Institute of Iran, Tehran, 1316943551 Iran; 2 Department of Nanobiotechnology, Pasteur Institute of Iran, Tehran, 1316943551 Iran; 3 Department of Biotechnology, Shahid Beheshti University, Tehran, 1983963113 Iran; 4 Department of Animal, Marine and Aquatic Biology and Biotechnology, Faculty of Life Sciences and Biotechnology, Shahid Beheshti University, Tehran, 1983963113 Iran; 5 Department of National Cell Bank of Iran, Pasteur Institute of Iran, Tehran, 1316943551 Iran; 6 Department of Cell and Molecular Sciences, Faculty of Life Sciences and Biotechnology, Shahid Beheshti University, Tehran, 1983963113 Iran

**Keywords:** Bio-adhesive material, MFP3, CsgA, DOPA, Augmented model predictions, Molecular simulation

## Abstract

Recombinant production of mussel foot proteins among marine-inspired proteinaceous adhesive materials has been attracted high attention for medical applications, due to their exceptional versatility potential of hierarchically arranged nanostructures. Various biochemical and proteinous factors such as amyloid CsgA curli protein have been used as a synergistic factor to enhance the constancy of obtained bio-adhesion but their mechanistic interactions have not yet been deeply investigated widely in different pH conditions. To this end, the present study has first sought to assess molecular simulation and prediction by using RosettaFold to predict the 3-dimensional structure of the fused CsgA subunit and the MFP3 protein followed by in vitro verification. It was developed an ensemble of quantitative structure-activity relationship models relying on simulations according to the surface area and molecular weight values of the fused proteins in acidic to basic situations using PlayMolecule (protein preparation app for MD simulations) online databases followed by molecular dynamic simulation at different pHs. It was found that acidic conditions positively affect adhesive strength throughout the chimeric structure based on comparative structure-based analyses along with those obtained in prevailing literature. Atomic force microscopy analysis was confirmed obtained in silico data which showed enhanced adhesive properties of fused protein after self-assembly in low pH conditions. In conclusion, the augmented model for reactivity predictions not only unravels the performance and explain ability of the adhesive proteins but in turn paves the way for the decision-making process for chimeric subunits modifications needed for future industrial production.

**Supplementary Information:**

The online version contains supplementary material available at 10.1186/s13568-022-01435-5.

## Introduction

Though numerous bio adhesive materials including marine derived proteins have been recently absorbed high attention to be used for medical purposes, their efficient and feasible production is not yet a reality owing to some environmental issues or complexity of the extraction from natural resources. Amongst marine organisms, mussels have a remarkable ability to attach to external surfaces by secreting a cluster of sticky proteins (Choi et al. 2011). These proteins located in the foot segment, the so-called mussel foot proteins (Waite 2017) are also known as adhesive plaque proteins due to their stability and permanent binding capacity (Lee et al. 2006). Concerning the classification, four genera of the *Mytilus* species were sorted according to the names *Mytilus californianus,* *Mytilus edulis,* *Mytilus trossulus,* and *Mytilus galloprovincialis* (*M.g*) (Myers et al. 2021). The surprising property of MFPs is to build molecular bonds between living organisms in excess of biocompatibility and biodegradability properties (Kaushik et al. 2015).

As a direct use of secretory mussel adhesion proteins, several thousand mussels are in demand for just 1 g of MFP protein extraction (Vareltzis et al. 2012) that certainly poses an ecological risk (Wu et al. 2010). The lower MFP content in mussels coupled with the poor performance of the extraction methods leads to an unsatisfactory extraction yield of MFP proteins from mussels (Castillo et al. 2017). On the other hand, a recent study has shown that none of the mussel populations was rated to hold good status in southeast Germany (Stoeckl et al. 2020). Given the above conditions, it is reasonably necessary to sequence and analyse natural *MFP* genes from different species of mussels in order to save them and make more efficient bioadhesives.

**Fig. 1:**
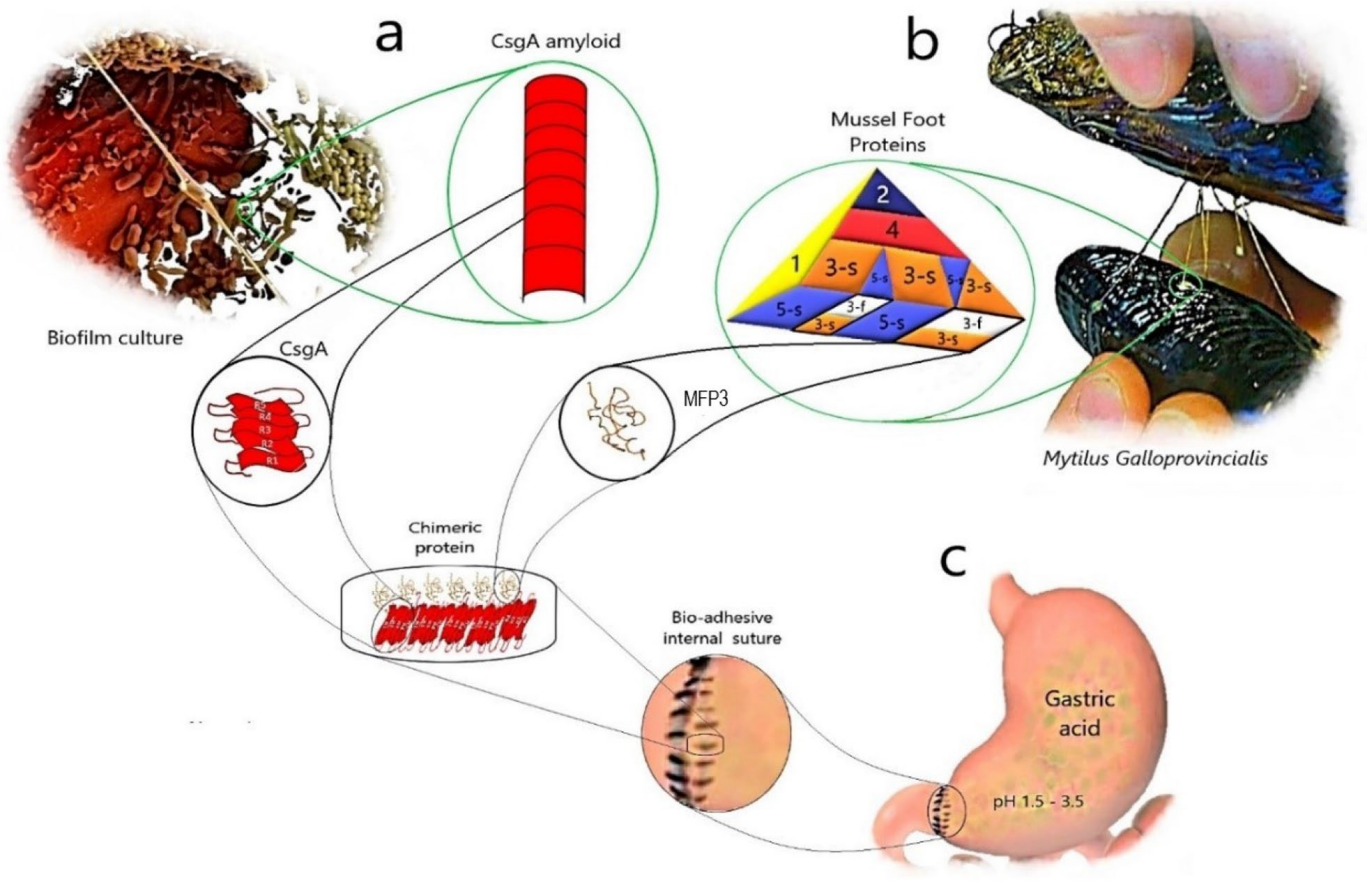
**a** A micrograph of mixed-culture biofilm. The secreted CsgA amyloid fibres allow the *E. coli* cells to collect to adhere to the surface. **b** *M.g* mussel and foot proteins distribution by number. MFP1 is already present in the outer layer as a lateral cover. MFP3 (s), MFP3 (f) and MFP5 (s) are shown at the plaque-substratum interface. The presence of MFP3 in both the substrate and the underlying layer considered it a good indicator to analyze its structure. **b** A suture with a bio-adhesive character on the gastric duodenum. The proteinous bio-adhesive construction, while making the connection between two sections, can maintain its biocompatible activity under the acidic conditions of the lumen

Previous studies in this area have been fairly advanced by in silico prediction and have been used in particular to measure adhesive force to demonstrate the intrinsic fluorescent activity of such bioadhesive chimeric proteins (Zhong et al. 2014). In addition, an older study indicates that lysine, threonine, tyrosine (Tyr), and 3,4-dihydroxyphenylalanine (DOPA) occupied 56% of all amino acid contents of similar adhesion proteins (Ohkawa et al. 1999) but these results refer to the amino acid percentage only as in vitro research. A graphical description of the process of this study is presented present to fully illustrate the sources of the chimeric protein, MFPs distribution in the adhesive plaque structure and its restorative uses (Fig. 1).

A non-replicating protein with an MW of 5–7 kDa has been identified as the smallest plaque protein in *Mytilus* mussel (Lin and Israelachvili 2007). The MFP3 itself is divided into two versions; MFP3(*f*) and MFP3(*s*) that offer their assembly speed (*fast* and *slow*). This protein is located in the middle and outer layer of the mussel foot plaque (Nicklisch et al. 2012) and contains 17–20 mol% and 8–14 mol% of the DOPA molecule for fast and slow versions, respectively (Wei et al. 2013). In the presence of the small size and highest abundance of glycine (18.2%) versus the MFP3 construction, it is more likely that the glycine will bend the protein structure (Jacob et al. 1999). Two famous protein versions (MFP3 and MFP5) have been observed in *Mytilus* plaque that has strong adhesion among all MFPs (Saha et al. 2020), which MFP3 also being a better option for subcloning, because of its lower molecular weight and more variants.

As the secondary domain of the chimeric protein, the CsgA is the largest helical subunit of a set of proteins that are actively secret by the bacterial cell (Van Gerven et al. 2015) Since the monomers of CsgA automatically curl into the helix molecular structure (Perov et al. 2019), the CsgA amyloid structure splits into singular repetitions of R1–R5 and 30% of the amino acids (Lembré et al. 2014). Molecular dynamic simulations of monomeric and fibrillary arrangements suggested that the amyloid formation of the CsgA core in cooperation with the MFP3 protein does not significantly disrupt the main amyloid structure (Cui et al. 2017). This finding means that the fused CsgA-MFP3 protein in all analysed samples has an amyloid-like structure relevant for the CsgA domains due to the most noticeable irregularity in MFP3 (due to the exposure to the amyloid core) (Zhong et al. 2014). But even the statistical evaluation of two of the studies mentioned above does not confirm the hypothesis about the protein surface area at different pH values. This work gives an overview of the most important alternatives in this area. Potential applications of mussel-inspired bio-adhesive proteins broadly cover the reconstructive industry, suture replacement (Bal-Ozturk et al. 2021), new tapes, polymers and medical devices (Saha et al. 2020), orthopaedics, dentistry and underwater facilities (Palacio et al.^2012^; Quan et al. 2019). By retaining the chimeric CsgA-MFP3 protein as an abdominal wound covering, surgical examinations such as vagotomy can be avoided (Yıldız et al. 2021; Roberts and Fitzpatrick. 2013).

We have recently achieved recombinant fused proteinous adhesive materials consist of mussel foot proteins, MFP3, MFP5, and GvpA and CsgA in both prokaryotic (*E. coli*) and eukaryotic expression systems (Irapour et al. 2021; Bolghari et al. 2022). In spite of inspiring properties and obtaining chimeric structure of obtained chimeric proteins, some obstacles are still needed to be solved prior to introduce a straightforward feasible approach for their industrial production. It has been reported that the mussels’ control dopa oxidation during adhesive plaque formation in their harsh natural niche by imposing an acidic and reducing regime. Thus, exploiting a mimetic bioinspired approach can be considered as a possible solution for enhanced adhesive strength of recombinant forms of proteins. Given the protein denaturation in acidic environments, one of the motivations behind this study is the biofriendly need of scientists to use adaptive structural and functional biomaterials under acidic conditions. The present study aimed to first generate in silico protein simulation and structure prediction using RosettaFold in addition to function prediction using displayed STRING and Cytoscape (PPi) followed by experimental validation in order to make a prolific synergy between theory and experimental science. Moreover, we will discuss the results of molecular docking and analysis by using PDBsum. The findings demonstrated a correlation between the in vitro and molecular simulation properties of the chimeric CsgA-MFP3 protein, providing the first suggestive evidence for surface analysis in the context of adhesive proteins.

## Materials and methods

### In silico study

#### Sequence analysis of the genes

The analysis of *MFP3* and *CsgA* genes was carried out using the EMBL and Genbank sequence database. The accession numbers for both genes of chimeric protein are provided in Table 1, separately.

**Table 1:** FASTA sequences of *MFP3* and *CsgA* genes in the Uniprot database

Gene	Chain	Amino acid sequence	Range	Accession number
MFP3	Signal peptide	MNNISVAVLVALVLIGSFAVQSDA	1–24	Q9GUX8
	Main	ADYYGPKYGPPRRYGGGNYNRYGR RYGGYKGWNNGWKRGRWGRKYY	25–70	
CsgA	Signal peptide	MKLLKVAAIAAIVFSGSALA	1–20	AY605712
	Main	GVVPQYGGGGNHGGGGNNSGPNSELNIYQYGGGNSALALQTDARNSDLTITQHGGGNGADVGQGSDDSSIDLTQRGFGNSALDQWNGKNSEMTVKQFGGGNGAAVDQTASNSSVNVTQVGFGNNATAHQY	21–151	

The synthesis of both genes was arranged without considering the signal peptide chain which includes 24 and 20 amino acids for *MFP3* and *CsgA* genes, respectively

#### DNA alignment and hybrid gene synthesis into the vector

To preserve the open reading frame (ORF) by the Snapgene software, it was ordered the synthesis of 600 base pairs hybrid genes into the pET28*-*(a) backbone vector, including *CsgA-GS linker-MFP3*. The recombinant *pET*-*28a (*+*)* vector was purified and the sequencing data was coordinated with the data from Biomatik Corporation (Ontario, Canada).

#### Molecular simulation and prediction of chimeric Mfp3-CsgA

The Robetta server was first used to determine the structure of chimeric mfp3-csgA. CAMEO is constantly evaluating Robetta as a protein structure prediction server. It can model multi-chain complexes using RoseTTAFold (the user must provide paired MSA) or comparative modeling (CM) and provides the option for large scale sampling (https://robetta.bakerlaboratory.org/).

#### Structural performance has been confirmed

The website (saves.mbi.ucla.edu) was used to authenticate the protein’s three-dimensional structure. The PDB format of the protein was submitted to PROCHECK, and a Ramachandran plot was subsequently generated.

#### Modification process

In order to carry out the modification process and to compare the resulting data with the unmodified version, it was decided to convert accessible Tyr amino acids of the entire protein structure into the l-DOPA. To this aim, since the simulation of the tyrosinase function on the substrate is linked to the number of free tyrosines (Roberts and Fitzpatrick. 2013), two strategies have been followed in PyMOL to determine available Tyr residues for tyrosinase activity (Fig. 3); 1, SA calculation, in which the surface area percentage for all tyrosines in the total protein construction was calculated; 2, A manual modification, in this scheme the number of available tyrosines facing the tyrosinase in the 3D structure of the chimeric protein counted by examining carbons number three and four from the benzene structure of each Tyr, which is the kind of confirmation of the first approach. Both methods have their merits and the choice depends on the particular study.

#### Molecular attaching

In terms of protein–protein docking capabilities, ClusPro’s web server is unmatched in the service sector. Protein–protein docking is possible using the ClusPro web server (https://cluspro.bu.edu/). PDBsum is an online database that contains statistics and derived docking results. Interacting with the display was accomplished using the discovery studio visualizer. FGF-2 is a growth factor for fibroblasts (PDB ID: 1DJS) It is a target of the fusion protein CsgA-MFP3.

#### Protein–protein interaction (PPI) network analysis

STRING is a database of known and predicted protein–protein interactions. The interactions include direct (physical) and indirect (functional) associations. The hub gene was determined using Cytoscape software (plugin Cytohubba).

#### Molecular dynamics

GROMACS is a type of molecular dynamics software that was developed largely for the purpose of simulating the behavior of lipids, proteins, and nucleic acids.

The three-dimensional (3D) structure of the principal curlin protein CsgA as complexes with foot protein 3 variant 3 was determined with the help of GROMACS version 2021.

### In vitro study

#### Cloning and expression of the CsgA-MFP3 hybrid gene in Rosetta

The coding sequences of the *CsgA* and *MFP3* genes including a *GS linker* were inserted between *XbaI* and *XhoI* restriction sites fused to a histidine-tag in the *pET-28a(* +*)* vector and synthesized (Biomatik Corp, Canada). To ensure correct insertion of CsgA-MFP3, polymerase chain reaction (PCR) was performed using primers listed in Table S1. Initially, Top10 competent cells were prepared using the calcium chloride procedure and the recombinant plasmid was transformed into the competent cells by the heat shock approach, then cultured overnight on a plate containing tetracycline for selection. A successful cloning process is guaranteed by the confirmation of the *Pst*I enzyme activity. The presence of fused genes in positive clones was validated by observing the colony-PCR and sequencing.

Reproduced bacteria from a single colony of the matrix culture were cultivated again in the LB medium and reached an amount of 0.5 of OD600, then after adding 100 mM IPTG after several hours of expression sampling was carried out.

#### SDS-PAGE, western blot and protein purification

After recording the OD600 values, adding appropriate amounts of SDS-PAGE loading buffer into the samples, boiling for 15 min, and centrifuging at 12,000×*g*, amounts of 10 μl of the individual case expressed from different hours are transferred into the 12% SDS-PAGE gel wells. According to the Western blotting protocol, after transferring to nitrocellulose paper and blocking with a blocking solution, the gel was treated with an HRP-supplemented polyhistidine antibody and washed by diluting 1:2000 in 1X PBS buffer, also the colour buffer for exposure bands, respectively. For protein purification, first, 50% of the Ni^2+^–NTA slurry (Qiagen) is pre-equilibrated on the supplement in ice-cold loading buffer, then a sufficient amount of 50% Ni^2+^–NTA slurry to bind the polyhistidine-tagged protein (5–10 mg/ml resin) was added and rotated at 4° for 1 h. After loading the resin into the column, it was washed with the loading buffer at 4° (same as the loading buffer but also containing 10 mM imidazole, pH to 8.0 with HCl). Then it was eluted with a gradient of 10 to 250 mM imidazole in loading buffer (pH up to 8.0 with HCl).

#### Tyrosinase treatment, amino acid and AFM analysis

In order to trap tyrosinase in the active site, the enzyme was soaked overnight in 1 mM of either CuSO_4_ and then in 1 mM of the appropriate ligand of the chimeric protein (l-Tyr). To ensure maximum occupancy, the ligand was soaked in various Tris–HCl buffers at pHs 3, 5, 7.4 and 9 for a further 1–5 min. For the precise determination of the DOPA/Tyr ratio, the amino acid analysis was carried out according to the HPLC method. Then the surface topography and mean average surface roughness were examined by atomic force microscopy (AFM) to correlate with the SA from in silico study. For this purpose, 20 µl of the modified chimeric protein including DOPA were tested in four different pH values and poured with 1 M acetic acid onto the mica surface and, after drying, placed in an AFM device.

## Results

### In silico results

#### Prediction of chimeric Mfp3-CsgA structure

**Fig. 2:**
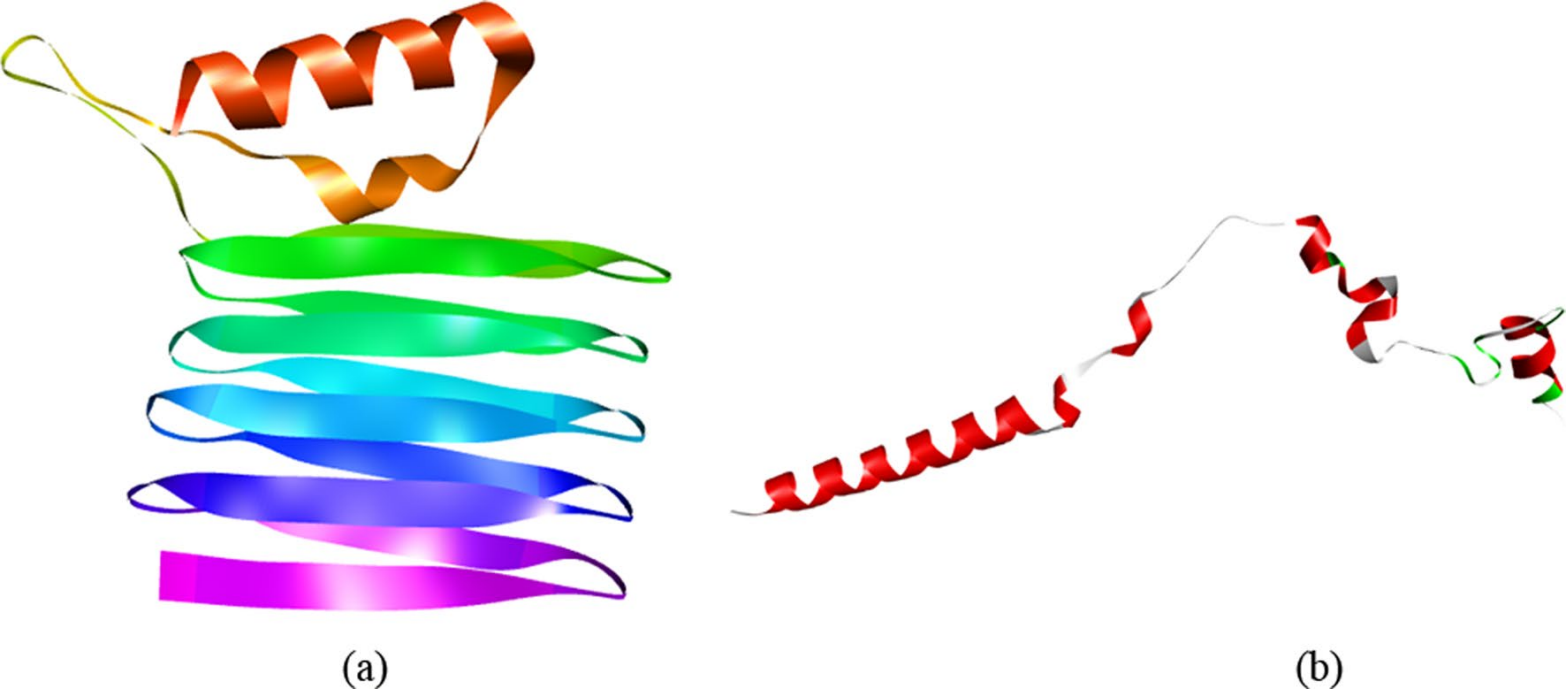
**a** Prediction RosettaFold surface atom and limits in Discovery Software were used to illustrate the three-dimensional structure of the chimeric CsgA-MFP3 protein. **a** Major curli protein CsgA, and **b** foot protein 3 variant 3.

The Rosetta server’s structure determination revealed that the fibril structure of the major curli protein CsgA was very accurate, and this server was also used to establish the structure of the foot protein 3 variation 3 (Fig. 2). Moreover interaction between two units of the fused protein was visualized using Pymol software (Additional file 1: Video S1).

#### Average molecular weight

**Fig. 3:**
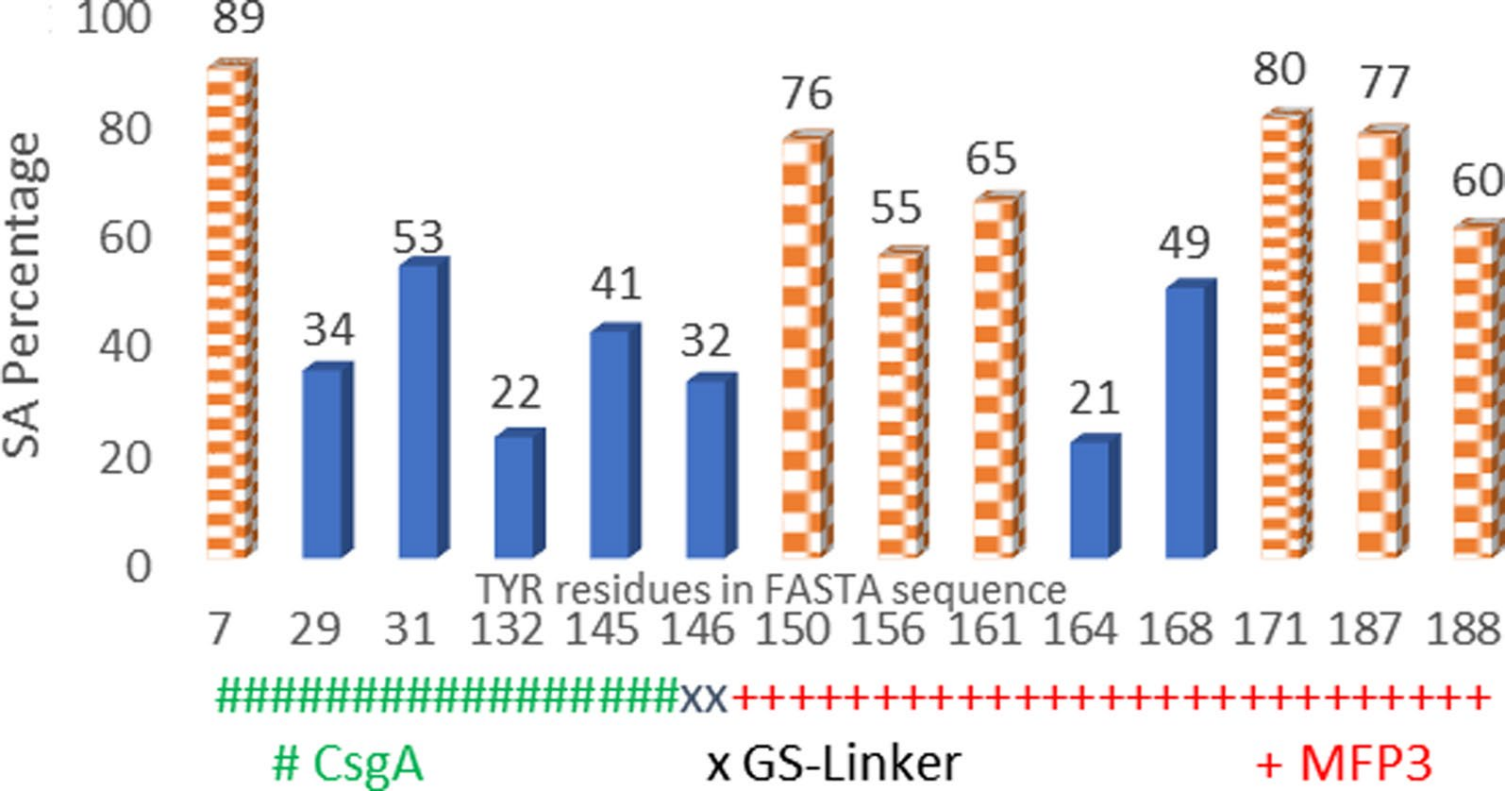
Data from the percentage of tyrosine surface area and determination of the manually modified by PyMOL. Manually modified Tyr residues with more than 55% of the quantified surface area. Unmodified tyrosine residues with less than 55% surface area. Six out of eight tyrosines in the MFP3 residue have been modified, which means that the tyrosine amino acids in the MFP3 structure are more accessible to tyrosinase activity than five unmodified tyrosines in the CsgA residue

The trend of the results shows that the pH values increase with increasing molecular weight. Modified samples are heavier than the unmodified ones. A plausible and useful theory behind the phenomenon is the maintenance of the OH molecules by Tyr residues. The pH analysis confirmed that the structure had acidic properties and the low pH increased activity and was able to motility (Additional file 1: Video S2). Mass spectrometry is a method for measuring the mass-to-charge ratio (m/z) of one or more molecules in a sample. These measurements are routinely used to determine the molecular weight of sample components (Fig. 3).

#### Molecular docking and validation of the structure

**Fig. 4:**
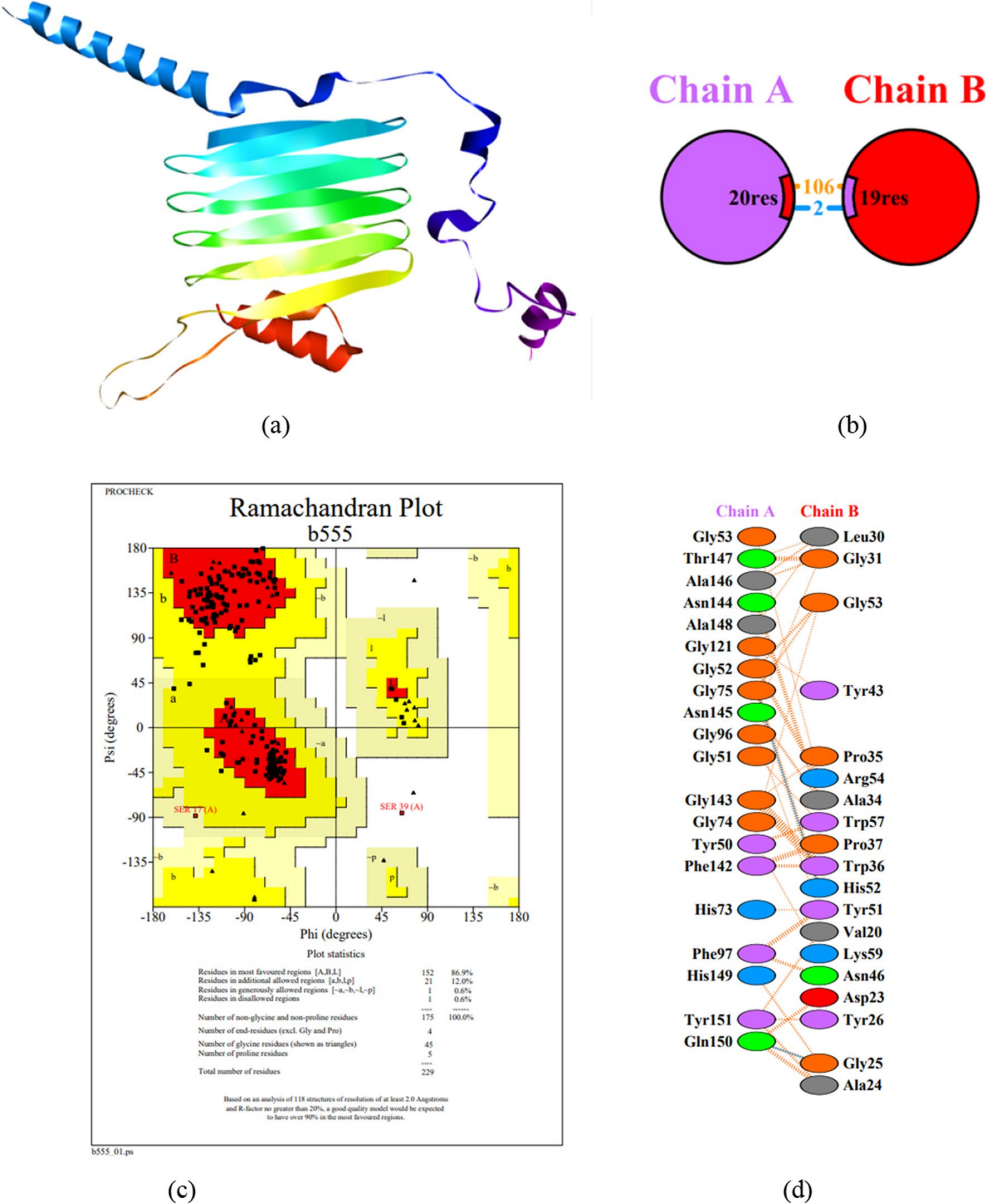
**a** Structure of chimeric protein. **b** Binding of two CsgA-mfp3 proteins to each other obtained by molecular docking using a Cluspro server. **b** Evaluation of docking results using the PDBsum server. **c** Ramachandran plot diagram. **d** Residues involved in the interaction of the chimeric protein Mfp3-csgA

**Fig. 5:**
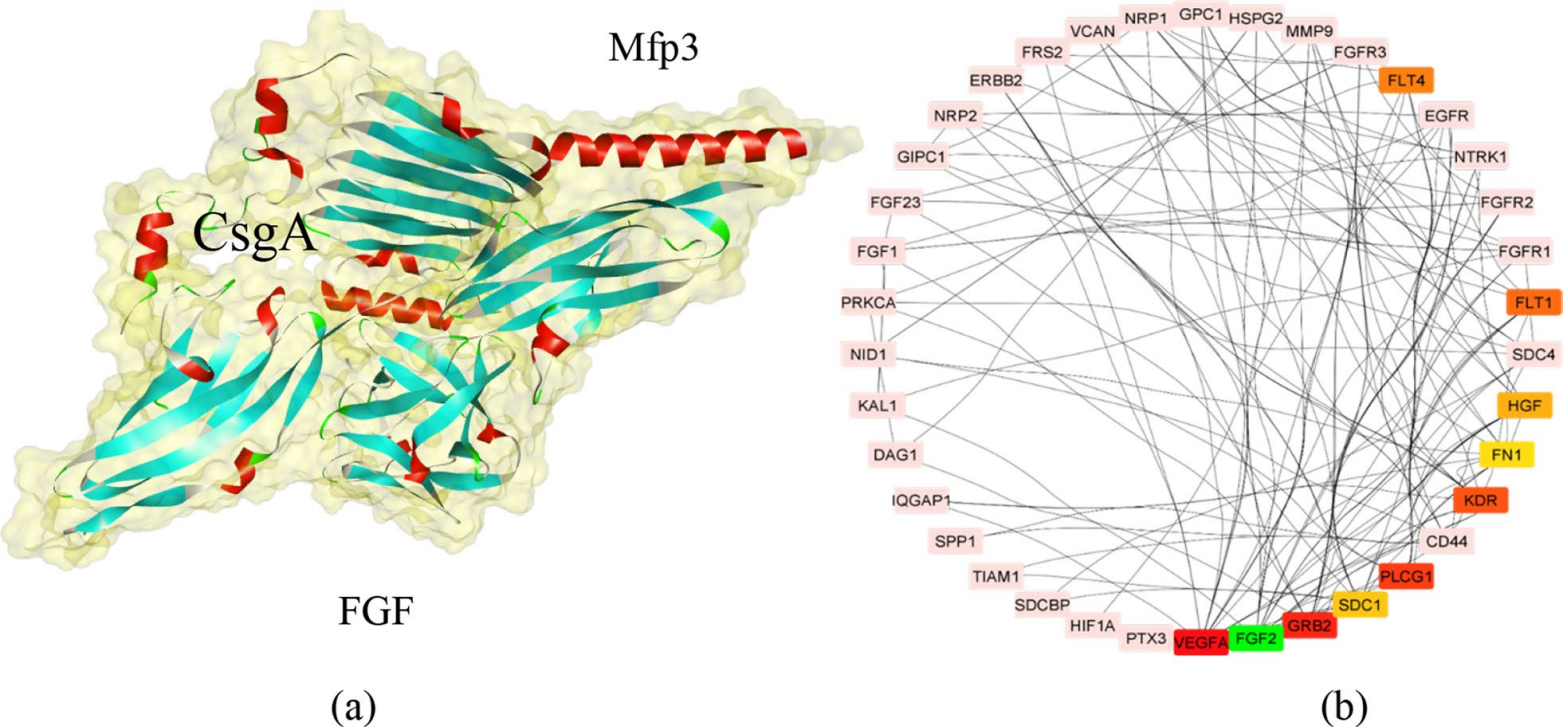
**a** Based on molecular docking studies, the predicted association between estimated and observed binding affinities for the complexation of chimeric CsgA-MFP3 to the fibroblast growth factors of the FGF-2 family is presented. A carton representation of complex CsgA-MFP3 and FGF2. The surface structure of the Molecular docking simulation that is being exhibited. Cylindrical representation of an atom and its limits. **b** Following the findings of clustering consisting of protein association networks of proteins using the cytoHubba plugin, one of the most popular sources of such networks is the Cytoscape software, which is one of the most widely used

Molecular modeling and prediction of the three-dimensional structure of the Mfp3-CsgA chimeric complex, as well as structural validation in the PROCHECK service. The fusion protein’s reflects the principle an exceedingly complicated fusion capable of improving network analysis. For example it interacts with the FGF2 protein (Fig. 4). Physical protein–protein interactions (PPIs) are highly specialized physical contacts formed between two or among more protein molecules as a consequence of biochemical activities that are guided by interactions such as electrostatic forces, hydrogen bonding, and the hydrophobic effect. Many are physical connections with molecular linkages among chains that occur in a cell or in a live creature in a particular biomolecular context, such as the environment in which the cell or organism is located (Fig. 5).

#### Molecular dynamics

**Fig. 6:**
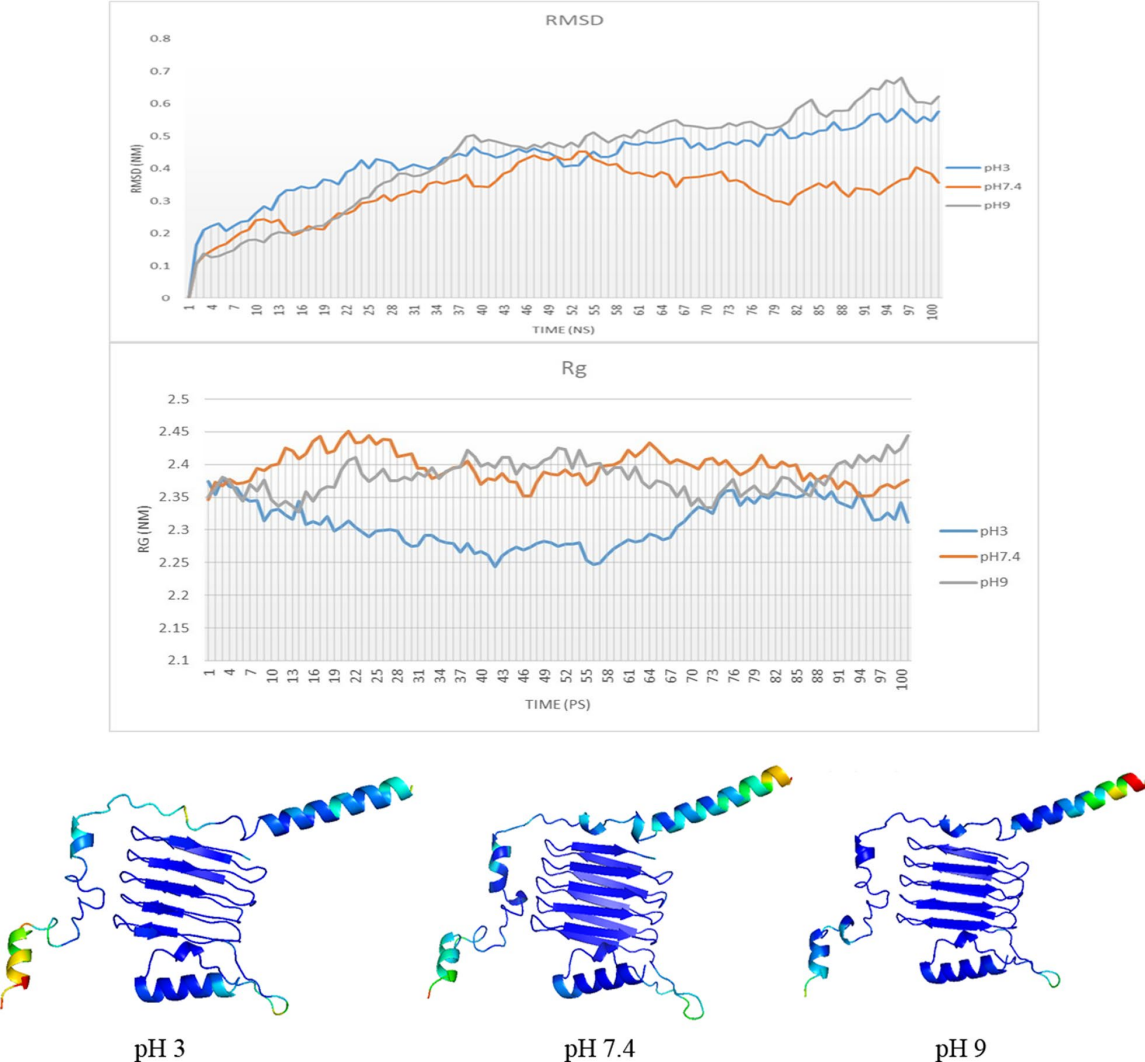
The value of the root mean square deviation (RMSD) for each atom is determined with reference to the primary structure, which is the reference structure. It does this by contrasting the primary structure with the secondary structure at any given time and illustrating the degree to which they have changed. Rg, A measurement of protein compaction that indicates the degree to which the structure is stable when simulated at varying pHs. Using factor B in Paymol software, flexible parts that have a lot of movement were identified. The presence of a second regular structure (beta sheets) reduces the rate of change and flexibility at different pHs, as shown by the blue regions, which indicates this reduction in motion. Red regions is associated with transition, movement and adaptation

Molecular dynamics simulation of the studied complex was performed using GROMACS software version 2021 and the selection of force field strength, CHRMM27, and water solvent model (spc) on the structure of chimeric protein mfp3-csgA became. The temperature used for this simulation is 300 K and the pressure is 1 bar. This simulation was performed in 1 ns. The minimized systems were submitted to two consecutive equilibration steps, considering a temperature of 300 K and a pressure of 1 bar, and with position restraint of the whole system, except for ions and water molecules. In the first step (1 ns), the number of particles, volume, and temperature were considered constant (NVT ensemble), and in the second step (1 ns), the system was considered isothermal-isobaric (NPT ensemble), (Fig. 6, and Video S2).

Inslico studies revealed the ability of Mfp3-CsgA chimeric proteins to interact. These proteins retain their good regular structure due to their acidic pH and folding base, and due to the flexibility of the CsgA protein, they have the ability to be chimeric for interaction with other proteins. At stable pH, this protein showed less flexibility (Fig. 6).

### In vitro results

#### Recombinant DNA alignment

CsgA-MFP3 chimeric construct were successfully cloned into the Rosetta strain of *E. coli* and expressed the desired fused protein. Sequencing data confirmed the complete nucleotide sequence of the gene inserted genes in transformed Rosetta strain (Additional file 1: Figure S1).

#### DNA analysis, SDS-PAGE, Western blot and protein purification

**Fig. 7.**
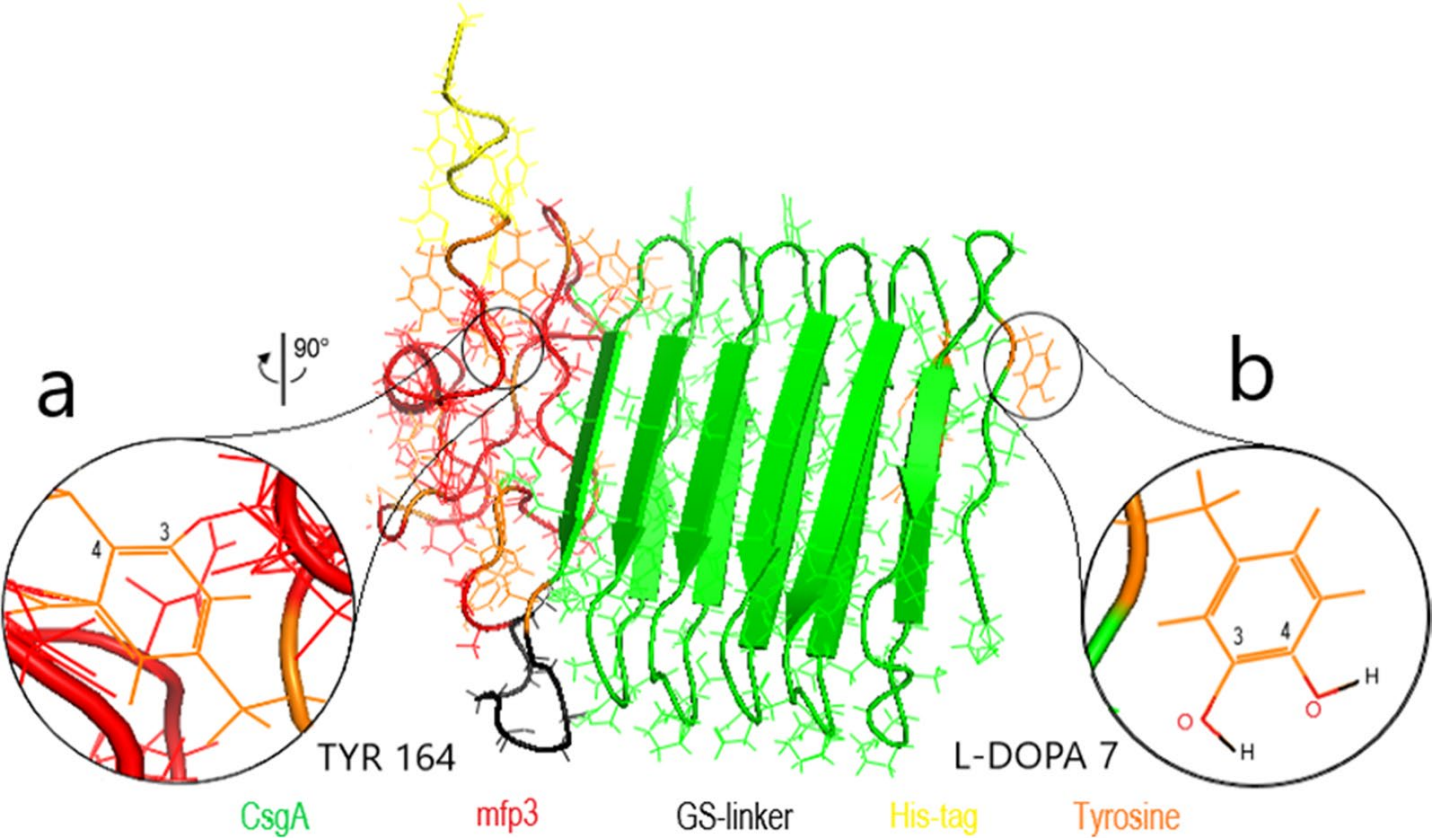
3D structure of the fused CsgA-MFP3 protein and manual modification in PyMOL. **a** A tyrosine involved in the MFP3 chain of the chimeric protein; The carbon numbers 3 and 4 of the benzene rings were bound to other internal amino acids and could not be counted as a tyrosinase substrate. **b** l-DOPA; a product of the tyrosinase enzymatic reaction on available Tyr#7

According to the 10–250 bp DNA and 10–180 kDa protein ladder sizes, the sample mass numbers 1–4 and 1–7 are displayed in the DNA electrophoresis gel and the SDS-PAGE gel, respectively. As it turns out, evidence of the desired protein expression from the obtained sample at the second hour of expression is presented. In addition, 1 mg of the protein was purified by Western blot assay and 1 mg of protein was finally purified using the His-tag antibody for the nickel column. The protein presence match results are shown (Fig. 7).

#### AFM analysis

The results of AFM analysis over a range of pH values appear reasonable and compatible with the software expectations (Fig. 7). A section of 25 μm^2^ at pH 9 shows the super-rough surface of the chimeric protein, the mean standard deviation which is amplified at 200 nm, while, according to the picture taken for the neutral pH range such as water environment, it can be observed that the surface is visibly smoother than under alkaline conditions. So that the highest peak is 200 nm. The 100 nm peak that belonging to the surface of the bio-adhesive at acidic pH 5, clearly explains the difference between its AFM image and the neutral environment. With an acidic pH, the surface is flatter than the neutral one. At pH 3, however, due to the oxidation process on DOPA, the surface of the structure is significantly wider than others, so that the maximum recorded peak does not exceed 40 nm, which indicates the special capability of the expressed bio adhesive to have more surfaces in a strongly acidic environments (around 10 nm), which by and large is clear that there is no simplistic explanation.

#### Amino acid analysis

**Fig. 8.**
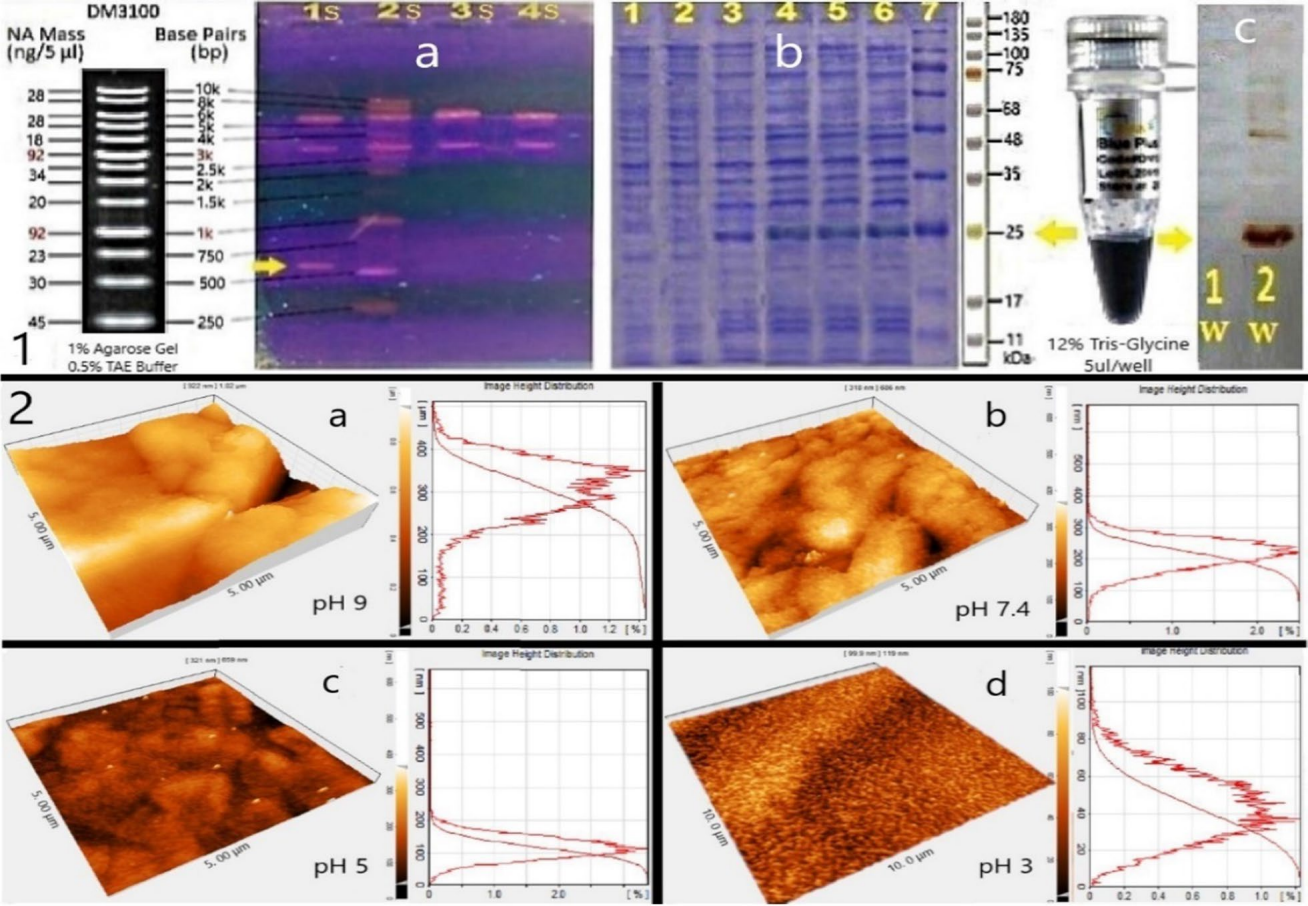
**1** Cloning and expression results. **a** Isolated DNA fragments by electrophoresis gel; 1S, colony PCR, 600 bp amplified DNA fragments belongs to the *CsgA-MFP3* hybrid gene; 2S, DNA ladder; 3S, *Pst*I restricted sample, plasmid cleavage within the *MFP3* gene made it standing very slightly above the backbone plasmid; 4S, backbone plasmid. **b** SDS-PAGE gel; 1, zero-hour* (negative control); 2, first hour; 3, second hour; 4, fourth hour; 5, sixth hour; 6, eighth hour; 7, marker; **c** Western blot gel; 1 W, zero-hour (negative control); 2 W, fourth hour; *, expression hour. **2** Surface topographies of the chimeric CsgA-MFP3 protein together with height distribution diagrams for individual pH values. A section of 25 μm^2^ at **a** pH 9, **b** pH 7.4, **c** pH 5, and **d** pH 3

The result of the repetitions for the tyrosine percentage determination with the HPLC method for the amino acid analysis is shown in Category 1 of Fig. 8. No error bars for manual analysis indicate the lack of an approximation for this method which only available reactive tyrosines are intended to convert DOPA into the 3D structure of the chimeric protein. Error bars related to the SD quantities (± 13.1 and ± 3.7 for amino acid analysis and ABCS method, respectively).

## Discussion

### Estimation of tyrosines modification per cent

According to the enzymological studies, the inactivity of the tyrosinase in the presence of Tyr-like amino acids such as phenylalanine confirms the catalytic capacity only on the tyrosine (Iranpour et al. 2021). Based on the calculated SA volumes and a manual analysis performed on individual tyrosine, the number of possible tyrosines converted to the DOPA molecules in the chimeric protein structure is 7 out of 14 (50%), which is closer to the percentage of the amino acid analysed by in vitro approach (Fig. 8) (Bal-Ozturk et al. 2021).

### Differences in molecular weight

Based on the measured molecular weight (MW) of the fused CsgA-MFP3 protein by in vitro method, the investigation of the relationship between MW and percent of modification was carried out in order to underpin the resulting curve from Fig. 3. Using the presented formula, it makes sense to explain the variations among molecular weight of samples (Eq. 1).

Equation 1 Equation of calculating MW difference between oxidized and non-oxidized amino acids. The *ow* is the oxygen atomic weight, *n* is the number of the oxygen added to the protein amino acids, *uw* belongs to the unmodified molecular weight, and *mw* refers to the modified molecular weight.

1 $$\left( {ow \times n} \right) + uw = mw$$

As in silico predictions for MW analysis, the influence of pH and modification on the molecular mass of the chimeric CsgA-MFP3 protein was measured. Under the acidic conditions, 14 OH molecules were bound to the protein to make a modified case. Since every single DOPA molecule carries a couple of OH groups, the difference in MW of OH molecules could be related to the modification of seven available tyrosine amino acids.

$$20.609 - 20.385 = 0.224{\text{ kDa}}$$

$$224 Da \div 16 \left( {ow} \right) = 14 \left( {OH} \right)$$

$$14 \left( {OH} \right) \div 2 = 7 \left( {DOPA} \right)$$

### Adhesion force and auto-oxidation

**Fig. 9:**
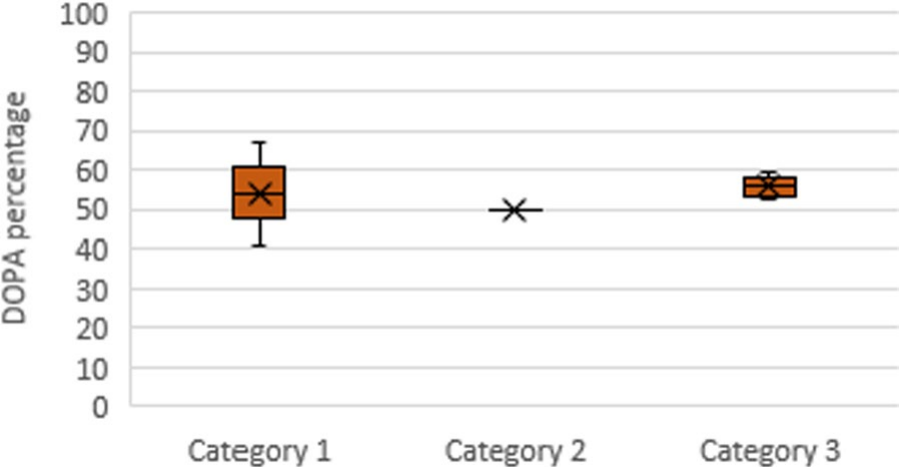
Comparison among different methods of modification determination. Category 1, amino acid analysis; Category 2, manual analysis; Category 3, ABCS method from a similar article with permission (Lin and Israelachvili 2007)

In order to measure the dependence of the adhesive force on the oxidation phenomenon, there is no adhesive bond with the DOPA quinone form (pH 7.4), in contrast to the DOPA structure at pH 3 (Fig. 9) (Castillo et al. 2017).

Regarding the auto-oxidation mechanism and the SA values of the modified versions in Fig. 9 for both pH values (acidic and neutral), the collected data show that there are approximately 20 Å^2^ differences in the SA from acidic (3) to close to the neutral pH (7.4) that indicates less exposure to DOPA, thereby reducing molecular expansion as a contribution factor is reducing the level of adhesion.

### Surface area, heigh distribution and adhesion

**Fig. 10.**
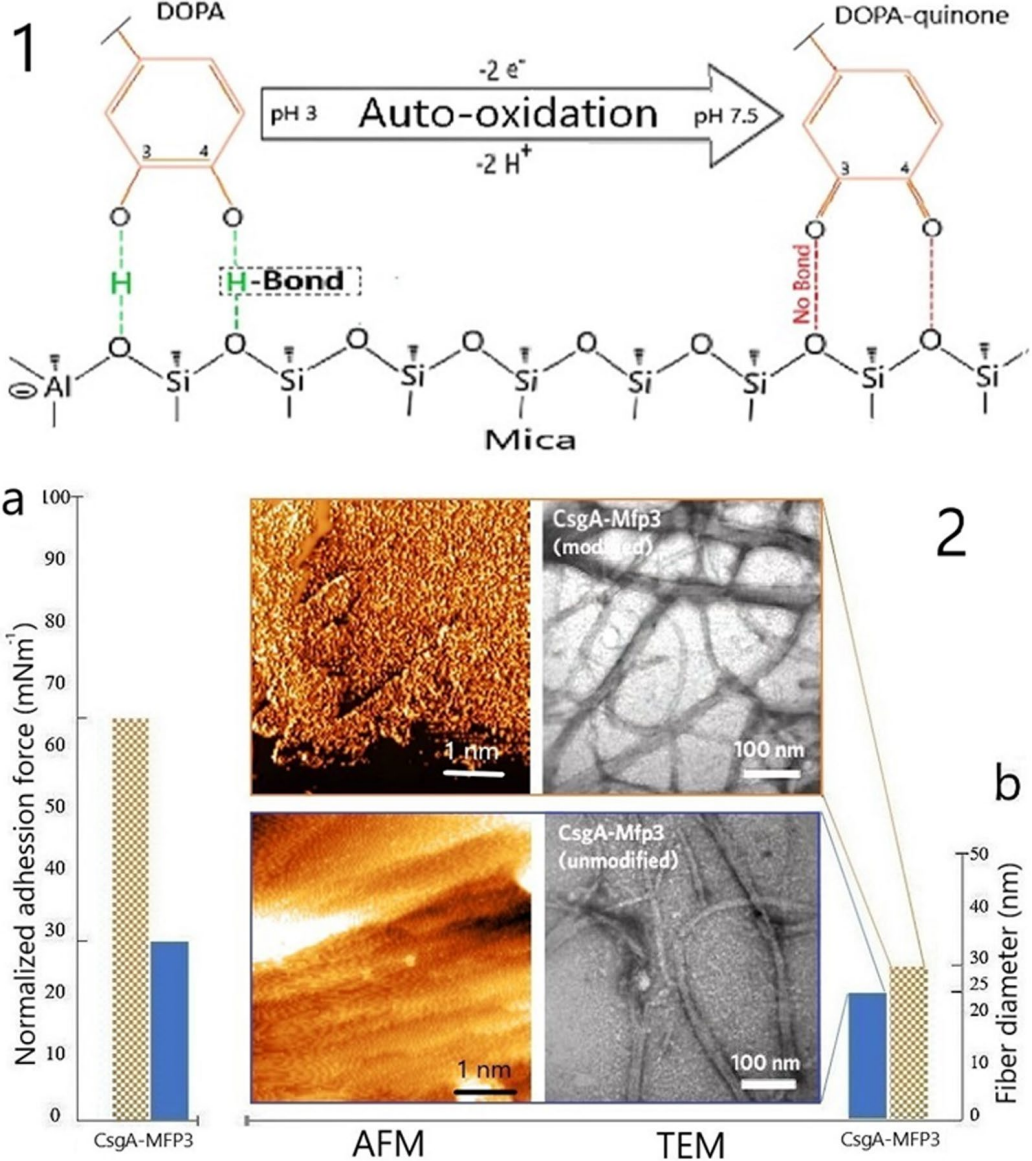
**1** Influence of auto-oxidation and pH scale on the formation of adhesive bonds. At pH 3, **a** more than twice the increase in the adhesive force of the modified case on the PS (polystyrene) surface by mNm^−1^ unit, compared to the unmodified version. **b** Size of the fibre diameter from AFM and TEM images for modified and unmodified versions of CsgA-MFP3 fibre after 3 days of incubation at 4 °C. There are important differences between unmodified and modified images of CsgA-MFP3 fibres based on approximately 5-nm differences between the amounts of fibre diameters

In the term of adhesion, the SA factor from in silico approach plays a contextual role for the adhesive force of the chimeric protein. Regardless of the direct correlation between SA and adhesion force, since the SA values for modified and unmodified cases do not justify a doubling of the adhesion force between them, but rather a seven-fold increase in DOPA presentation (indicating the qualitative role of DOPA instead of the quantity). As in vitro studies from AFM tests have shown, the adhesive strengths correspond more to the gradual decrease in the height distribution in the individual stages of the pH decrease. The height distribution data from the AFM test, along with dependent plots, explain well the effect of SA accumulation for each individual protein on the ultimate fibril formation (Choi et al. 2011). A steady increase in the pH scale leads to the fact that a DOPA-quinone structure is created through auto-oxidation which cannot form an adhesive bond so that the modified samples on a high pH scale become similar to the unmodified cases. This manner could be seen as a relevant reason for a significantly weaker adhesive force at pH 9–10 for similar chimeric proteins such as CsgA-MFP5 (Zhong et al. 2014). The result of changes in the fibre diameter could be derived from the SA of the DOPA structure in the modified samples compared to the unmodified without DOPA. The OH groups on carbons atoms number 3 and 4 of benzene are stable, which provides a suitable surface for binding to the mica. In contrast to this, the DOPA structure could lose a few H^+^ molecules through to the auto-oxidation process at pH 7.5, which can create double bonds between the oxygen and the specific carbons, so that there are no hydrogen bonds are formed to the mica surface, as a DOPA quinone form (Fig. 10).

Altogether, present study exploited in silico approaches to confirm the performance of the co-expression system for fused CsgA and *Mytilus* MFP3 protein. In addition, molecular dynamics simulations predicted the 3D structure of the fused CsgA-MFP3 protein as well as PyMOL’s determination of the SA estimate and manual modification methods that predict the percentage and finality of each Tyrosine modification, respectively (Additional file 1: Videos S1). In addition, in silico studies have carefully predicted that the strength of adhesion depends on both the SA and DOPA levels of the fusion protein, which could be deduced from an unusual difference between pH 7.4 and 9 of the modified and unmodified versions. The minimal difference between the SA contents of modified and unmodified samples may not be the main reason for the double increase in the adhesion energy in Fig. 9a, but the currently unexpected tendency and the loss of adhesive strength in the alkaline range is a compelling reason for finding a meaningful relationship between SA and adhesion force factors. As expected, in vitro and in silico strategies could explain the maintenance of chimeric protein adhesion under strongly acidic conditions such as gastric lumen pH 3 (Cui et al. 2017), which represents a worthy advance in the area of tissue binding. The SA levels of DOPA as the primary agent in the adhesion process were estimated, whereby the role of such parameters became clearer through the lowering of the pH scale, while in the fibrillar view of the structure, the height distribution factor together with the real image display provided more relevant relationships. Therefore, the lower adhesive force in alkaline situations is influenced by the reduction in the DOPA surface, which, corresponds to the lower surface area, due to the higher height distribution.

## Supplementary Information

**Additional file 1: Table S1.** List of used primers. **Table S2. **The binding score describes extremely specific physical connections generated between two or more protein molecules as a result of biochemical reactions led by electrostatic forces, hydrogen bonding, and the hydrophobic effect.** Figure S1.** Sequencing analysis of inserted genes in transformed clones. **Video S1.** Structure Model of fused protein. The animation of the chimeric protein model was presented using Pymol software to better illustrate the interaction between proteins to better visualize the different angles of the protein complex in the reader’s mind. **Video S2.** Molecular Dynamics Simulations at pH3. Visualization of how a protein complex behaves at an acidic pH is one of the most important achievements of the analysis in computational conditions, a process that is not observable in the laboratory. As shown in molecular dynamics analysis, protein folding is maintained at acidic and base pH due to the regular structure of protein Mfp3, which can exhibit extremely strong performance.

## Data Availability

The dataset related to the synthesized nucleotide sequences synthesis in current study have registered and are available on the NCBI database under accession no. Banklt2575895 On338069.
