# Supplementary material for: In silico prediction and in vitro validation of the effect of pH on adhesive behaviour of the fused CsgA-MFP3 protein
Source: AMB Express. 2022 Jul 15;12:94. doi: 10.1186/s13568-022-01435-5 (PMC9287526; doi:10.1186/s13568-022-01435-5)
Supplement: Supplementary file 1 — Additional file 1: Table S1. List of used primers. Table S2. The binding score describes extremely specific physical connections generated between two or more protein molecules as a result of biochemical reactions led by electrostatic forces, hydrogen bonding, and the hydrophobic effect. Figure S1. Sequencing analysis of inserted genes in transformed clones. Video S1. Structure Model of fused protein. The animation of the chimeric protein model was presented using Pymol software to better illustrate the interaction between proteins to better visualize the different angles of the protein complex in the reader’s mind. Video S2. Molecular Dynamics Simulations at pH3. Visualization of how a protein complex behaves at an acidic pH is one of the most important achievements of the analysis in computational conditions, a process that is not observable in the laboratory. As shown in molecular dynamics analysis, protein folding is maintained at acidic and base pH due to the regular structure of protein Mfp3, which can exhibit extremely strong performance. [file 13568_2022_1435_MOESM1_ESM.zip › Supplementary Materials.doc]

**Additional file 1.**

**Table S1. List of used primers**

| Application | Primer | Sequence | Row |
| --- | --- | --- | --- |
| Gene existence | Left (SEQ_L1) | 5′-ATGGCGGCGGCGGCAATAAT-3′ | *1* |
|  | Right (SEQ_R1) | 5′-AACGACCGCGTTTCCAGCCA-3′ | *2* |

Note: The melting temperatures (Tm) were 59.16 °C and 59.56 °C for the left and right primers, respectively, and 60% CG content for both, calculated on the GeneScript online database.

**Table S2.** The binding score describes extremely specific physical connections generated between two or more protein molecules as a result of biochemical reactions led by electrostatic forces, hydrogen bonding, and the hydrophobic effect.

| **Cluster** | **Members** | **Represented** | **Weighted** |
| --- | --- | --- | --- |
| 0 | 50 | Centre | -1219.9 |
| 0 | 50 | Lowest En | -1305.9 |
| 1 | 40 | Centre | -1227.4 |
| 1 | 40 | Lowest Energy | -1434.9 |
| 2 | 35 | Centre | -1204.7 |
| 2 | 35 | Lowest Energy | -1375.2 |
| 3 | 35 | Centre | -1145.9 |
| 3 | 35 | Lowest Energy | -1304.6 |
| 4 | 28 | Centre | -1225.3 |
| 4 | 28 | Lowest Energy | -1293.6 |
| 5 | 25 | Centre | -1088.5 |
| 5 | 25 | Lowest Energy | -1357.4. |
| 6 | 24 | Centre | -1176.9 |
| 6 | 24 | Lowest Energy | -1197.4 |
| 7 | 22 | Centre | -1104.1 |
| 7 | 22 | Lowest Energy | -1289.7 |
| 8 | 21 | Centre | -1154.1 |
| 8 | 21 | Centre | -1317.2 |
| 9 | 21 | Lowest Energy | -1164.1 |
| 9 | 21 | Centre | -1368.5 |
| 10 | 21 | Lowest Energy | -1309.1 |
| 10 | 21 | Centre | -1309.1 |

**Figure S1.** Sequencing analysis of inserted genes in transformed clones.

**Video S1. *Structure Model Mp4***

The animation of the chimeric protein model was presented using Pymol software to better illustrate the interaction between proteins to better visualize the different angles of the protein complex in the reader's mind.

**Video S2. *Molecular Dynamics Simulations Mp4 pH3***

Visualization of how a protein complex behaves at an acidic pH is one of the most important achievements of the analysis in In silico conditions, a process that is not observable in the laboratory. As shown in molecular dynamics analysis, protein folding is maintained at acidic and bases pH due to the regular structure of protein Mfp3, can exhibit extremely strong performance.
